# A Cross-Sectional Study on the Prevalence and Predictors of Cognitive Impairment and Depression in Elderly Patients With Type 2 Diabetes Mellitus

**DOI:** 10.7759/cureus.77753

**Published:** 2025-01-21

**Authors:** Fatima Khan, Sartaj Hussain, Somya Singh, K K Sawlani, Kauser Usman, Amod K Sachan, Sanjay Khattri

**Affiliations:** 1 Pharmacology, Dr. Ram Manohar Lohia Institute of Medical Sciences, Lucknow, IND; 2 Pharmacology, All India Institute of Medical Sciences (AIIMS) Jammu, Vijaypur, IND; 3 Pharmacology, All India Institute of Medical Sciences (AIIMS) Bhopal, Bhopal, IND; 4 Internal Medicine, King George's Medical University, Lucknow, IND; 5 Pharmacology and Therapeutics, King George's Medical University, Lucknow, IND

**Keywords:** aging, cognitive impairment, depression, elderly, type 2 diabetes mellitus

## Abstract

Background

The rising prevalence of type 2 diabetes mellitus (T2DM) in the elderly is associated with mental health disorders like cognitive impairment and depression due to hyperglycemia and inflammation. The present study aims to estimate the prevalence of cognitive impairment and depression and its association with clinical, biochemical, and psychosocial factors to identify high-risk subjects.

Methods

A cross-sectional study was conducted at a tertiary care teaching hospital and enrolled 99 patients of T2DM, aged equal to or more than 60 years from North India. Cognitive function and depression were assessed by the Hindi Mental Scale Examination (HMSE) and the Hindi version of the Geriatric Depression Scale (GDS-H), respectively. Fasting plasma glucose (FPG), glycated hemoglobin (HbA1C), and the lipid profile were measured. Univariate and multivariate binary logistic regression analyses were applied to identify association and independent predictors, respectively, and receiver operating characteristic (ROC) curve analysis to determine the optimal cut-off values.

Results

The mean age of the patients was 66.68 years, and 38.38% were females. The prevalence of impaired cognition and depression in elderly T2DM subjects was 37.37% and 43.43%, respectively. HMSE was inversely correlated while GDS-H was positively correlated with FPG and HbA1C. Cognitive impairment was independently predicted by age, HbA1C levels, and rural residence. Depression was independently associated with HbA1C levels and being unmarried or widowed. The optimal cut-off for cognitive impairment was age >63 years and HbA1C >7.7%. For depression, the cut-off for HbA1C was >6.9%.

Conclusion

This study revealed a higher prevalence of cognitive impairment and depression among elderly T2DM patients in North India. The age of more than 63 years, poor glycemic control, rural residents, and marital status are high-risk groups for cognitive impairment and depression. This study suggests the integration of routine mental health screening for high-risk elderly T2DM patients and the development of comprehensive diabetes management programs that address both physical and mental health aspects.

## Introduction

Type 2 diabetes mellitus (T2DM) is a public health challenge characterized by hyperglycemia and insulin resistance. As the world population ages, the prevalence of T2DM is increasing among the elderly [1]. In the long term, T2DM not only causes microvascular and macrovascular complications but also affects cognitive and psychological health [2]. T2DM can be considered an accelerated model of aging [3]. Aging makes the elderly prone to cognitive impairment and depression, and the presence of T2DM further exacerbates these conditions. The presence of cognitive impairment and depression in T2DM is a significant challenge, as it reduces quality of life, increases morbidity, and needs long-term care [4].

Chronic hyperglycemia in T2DM leads to microvascular complications, oxidative stress, and neuroinflammation, which result in neurotoxicity. These processes trigger neurodegenerative changes in diabetes and make T2DM individuals more vulnerable to cognitive dysfunction and mental disorders [5]. Psychosocial factors, such as education status, marital status, rural vs urban living conditions, and socioeconomic status, are key determinants of cognitive function and mental health [6,7]. A meta-analysis reported that the prevalence of depression in the T2DM population was 28% globally and 32% in Asia [8]. In another meta-analysis, the prevalence of mild cognitive impairment in individuals with T2DM was reported to be 45% [9].

This study aims to estimate the prevalence of cognitive impairment and depression and its association with clinical, biochemical, and psychosocial factors in elderly T2DM patients who have never received a formal diagnosis of cognitive impairment and depression. This study also determines the independent predictors of cognitive impairment and depression to identify high-risk individuals in the North Indian elderly T2DM population. Determining the prevalence and predictors of cognitive impairment and depression in elderly T2DM patients will help identify high-risk subjects, guide target screening, and develop comprehensive diabetes management programs.

## Materials and methods

General study settings

This cross-sectional study was conducted in the Department of Pharmacology in collaboration with the Department of Medicine of King George's Medical University, Lucknow. Patients who had never been diagnosed with cognitive impairment or depression were screened based on the selection criteria. A total of 99 patients aged 60 years and above with type 2 diabetes mellitus, as defined by the International Diabetes Federation (IDF) 2017 guidelines [10], were enrolled. Patients with type I diabetes mellitus, dementia, depression, a history of psychiatric disease, cognitive impairment, any coexisting neurological disease like Alzheimer’s disease, Parkinson’s disease, and cerebrovascular diseases, such as stroke, complicated hypertension, renal failure, genetic disorder, HIV disease, and any kind of cancer were excluded. The study was approved by the institutional ethics committee (Ref. code: 102nd ECM II B- Thesis/P44). Written informed consent was taken from all participants. The case history was recorded through questionnaires and personal interviews. In the study, rural and urban populations were defined based on India's administrative classification. Rural areas fall under Gram Panchayats while urban areas are governed by Municipalities.

Biochemical analysis

Patients were called after overnight fasting, and 5 ml of venous blood was drawn from the antecubital vein by a standard venipuncture method and divided into three parts. One part (1.5 ml) was kept in a fluoride vial for fasting glucose estimation, the second part (1.5 ml) was kept in a K3 EDTA vial, and the third part (2 ml) was kept in a plain vial. Fasting plasma glucose and lipid profile were determined using the 'SELECTRA' auto-analyzer (PRO XL) and related kits. Glycosylated hemoglobin (HbA1C) was quantified by 'BIO-RAD' D-10TM high-performance liquid chromatography (HPLC) and a related kit.

Sample size calculation

The sample size was calculated by n = (1.96)2 × p×(1-p)/d2, where p is prevalence and d is the margin of error. In a study by Solanki et al. (2009), they reported that the prevalence of impaired cognition was 48% in elderly diabetic patients [11]. Taking a prevalence of 48% of poor cognition, assuming 80% power, a 5% significance level with a 95% confidence interval, and a margin error of 10%, the sample calculated was 96.

Evaluation of cognition

Cognition was ascertained using the Hindi Mental Scale Examination (HMSE) [12]. The HMSE is a validated version of the Mini-Mental State Examination (MMSE) for Hindi language persons developed especially for the illiterate elderly Indian population. The HMSE has a total score of 30 and is categorized into no cognitive impairment (score of 24 or more) and cognitive impairment (score of less than 24).

Evaluation of depression

The Hindi version of the Geriatric Depression Scale (GDS-H) was used to assess the depression [13]. In the GDS-H, there is a 30-question scale, and each question has a binary response of ‘yes’ or ‘no,’ with a score of 0 or 1, resulting in a total score of 30. The score is categorized into normal, mild depression, and severe depression, with a score of 0-9, 10-19, and 20-30, respectively.

Statistical analysis

The data were checked for distribution using the Shapiro-Wilk test. All variables were normally distributed except for the Geriatric Depression Scale (GDS-H). Results are presented as mean ± SD, median (interquartile range), or number (percentages). The normally distributed parameters were compared with the unpaired student's t-test. The GDS-H was compared by the Mann-Whitney U test and represented as the median (interquartile range). Correlation between the variables was done by applying Spearman correlation analysis. For binary logistic regression, HMSE was dichotomized to normal (score ≥ 24) and cognitive impairment (score < 24), and GDS-H was dichotomized to normal (score: 0-9) and depression (score: 10-30). Univariate binary logistic regression analysis was applied to calculate the odds ratios for depression and cognitive impairment. Variables with P value ≤ 0.10 in the univariate binary logistic regression analysis were used in multivariate binary logistic regression by the forward conditional method to determine the independent predictors for depression and impaired cognition. The receiver operating characteristic (ROC) curve analysis was performed on those continuous variables that were independent predictors of impaired cognition and depression in the multivariate binary logistic regression. The optimal cut-off values were determined by the Youden index. Statistical analyses were performed in SPSS version 25.0 software for Windows (IBM Corp., Armonk, NY, USA). Two-sided P<0.05 was considered statistically significant.

## Results

A total of 99 patients with type 2 diabetes mellitus were recruited in the study. The demographic, clinical, and biochemical parameters are shown in Table 1. The mean age of patients was 66.68 years, and females were 38.38% of the total. The mean age at diagnosis of diabetes was 54.66 years, and the mean duration of T2DM was 11.9 years. The 46.46% of T2DM patients had a positive family history. Among glycaemic indices, the fasting plasma glucose (FPG) was 149.59 mg/dl, and HbA1C was 8.2%. The total cholesterol (TC), triglycerides (TG), high-density lipoprotein-cholesterol (HDL-C), and low-density lipoprotein-cholesterol (LDL-C) were calculated as 140.88, 162.04, 44.03 and 64.44 mg/dl, respectively. The mean HMSE and median GDS-H scores were 24.61 and 7.00, respectively. The prevalence of impaired cognition in elderly T2DM subjects was 37.37%. The prevalence of depression was 43.43%, with 14.14% classified as mildly depressed and 29.29% as severely depressed.

**Table 1:** Demographic, clinical, biochemical, and psychosocial characteristics of type 2 diabetes mellitus patients Data is represented as mean±SD, median (interquartile range), or number (%). BMI: Body Mass Index, FPG: Fasting Plasma Glucose, GDS-H: Geriatric Depression Scale-Hindi, HbA1C: Glycated Hemoglobin, HDL-C: High-Density Lipoprotein-Cholesterol, Hindi Mental State Examination: HMSE, LDL-C: Low-Density Lipoprotein-Cholesterol, TC: Total Cholesterol, TG: Triglyceride

Parameters	
Age (years)	66.68±5.56
Male/Female	61(61.62%)/38(38.38%)
Age at Diagnosis of Diabetes (years)	54.66±8.01
Duration of Diabetes (years)	11.9±7.12
Family History of Diabetes (Yes/No)	46(46.46%)/53(53.54%)
Hypertension (Yes/No)	64 (64.6%)/35 (35.4%)
BMI (kg/m^2^)	26.75±5.18
Systolic Blood Pressure (mmHg)	143.23±16.36
Diastolic Blood Pressure (mmHg)	84.31±11.22
FPG (mg/dl)	149.59±61.94
HbA1C (%)	8.2±1.95
TC (mg/dl)	140.88±43.09
TG (mg/dl)	162.04±64.57
HDL-C (mg/dl)	44.03±11.01
LDL-C (mg/dl)	64.44±36.59
Diet (Veg/Nonveg)	50(50.50%)/49(49.50%)
Smoking (Yes/No)	37(37.37%)/62(62.63%)
Drinking (Yes/No)	16(16.16%)/83(83.84%)
Religion (Muslim/Hindu)	19(19.19%)/80(80.81%)
Residence (Urban/Rural)	67(67.68%)/32(32.32%)
Employed/ Unemployed/ Retired	27(27.27%)/37(37.37%)/35(35.35%)
Social Status	
Upper (I)/Upper Middle (II)/Lower Middle (III)/Upper Lower (IV)/Lower (V)	0 (0%)/28 (28.28%)/35 (35.35%)/36 (36.36%)/0 (0%)
Education (literate/Illiterate)	81(81.81%)/18(18.19%)
Married/ Widow & Unmarried	88(88.89%)/11(11.11%)
Hindi Mental State Examination (HMSE)	24.61±3.2
Normal Cognition (HMSE ≥24)/Impaired Cognition (HMSE <24)	62 (62.63%)/37 (37.37%)
Geriatric Depression Scale (GDS-H)	7.00 (2.00-20.00)
Normal (0–9)/Mild Depression (10–19)/Severe Depression (20–30)	56 (56.57%)/14 (14.14%)/29 (29.29%)

Table 2 shows the HMSE and GDS-H scores, as well as the prevalence of impaired cognition and depression across various groups. The HMSE score was significantly lower in the rural as compared to the urban population. The median GDS-H score was significantly higher in the female and uneducated groups. The prevalence of impaired cognition was significantly higher in rural individuals; however, the prevalence of depression was higher in the female group but it did not reach a statistically significant level. Illiterate and unmarried & widowed individuals had a higher prevalence of depression.

**Table 2:** GDS-H score, HMSE score, prevalence of depression, and impaired cognition in various groups Statistically significant values are shown in bold. HMSE and GDS-H are presented as mean±SD (compared by the unpaired student’s t-test) and median (interquartile range) (compared by the Mann-Whitney U test). Categorical data are compared by the chi-square test. A two-tailed p < 0.05 is considered significant. GDS-H: Geriatric Depression Scale-Hindi, Hindi Mental State Examination: HMSE

Gender			
	Male (n=61)	Female(n=38)	P value
GDS-H	4.00 (1.00-19.00)	12 (6.5-22.00)	0.001
Normal/Depression	39(63.9%)/22(36.1%)	17(44.7%)/21(55.3%)	0.061
HMSE	24.61±3.29	24.61±3.1	0.998
Normal/Impaired cognition	36(59.0%)/25(41.0%)	26(68.4%)/12(31.6%)	0.347
Residence			
	Urban (n=67)	Rural (n=32)	P value
GDS-H	7.00 (1.00-20.00)	11.00 (5.00-22.00)	0.032
Normal/Depression	41(61.2%)/26(38.8%)	15(46.9%)/17(53.1%)	0.179
HMSE	25.23±3.11	23.28±3.02	0.004
Normal/Impaired cognition	48(71.6%)/19(28.4%)	14(43.8%)/18(56.3%)	0.007
Employment			
	Employed (n=27)	Unemployed & retired (n=72)	P value
GDS-H	8.00 (2.00-14.00)	7.00 (2.00-21.75)	0.503
Normal/Depression	16(59.3%)/11(40.7%)	40(55.6%)/32(44.4%)	0.741
HMSE	24.22±3.28	24.75±3.19	0.468
Normal/Impaired cognition	15(55.6%)/12(44.4%)	47(65.3%)/25(34.7%)	0.373
Education			
	Literate (n=81)	Illiterate (n=18)	P value
GDS-H	7.00 (1.00-20.00)	16.50 (6.5-22.25)	0.011
Normal/Depression	50(61.7%)/31(38.3%)	6(33.3)/12(66.7%)	0.028
HMSE	24.89±3.11	23.33±3.38	0.062
Normal/Impaired cognition	54(66.7%)/27(33.3%)	8(44.4%)/10(55.6%)	0.078
Marital Status			
	Married (n=88)	Unmarried & Widowed (n=11)	P value
GDS-H	7.00 (2.00-20.00)	19.00 (14.00-22.00)	0.138
Normal/Depression	54(61.4%)/34(38.6%)	2(18.2%)/9(81.8%)	0.006
HMSE	24.74±3.19	23.55±3.24	0.246
Normal/Impaired cognition	57(64.8%)/31(35.2%)	5(45.5%)/6(54.5%)	0.212

The correlation of various parameters with the HMSE and GDS-H score using Spearman’s rank correlation is shown in Table 3. The HMSE was found to be significantly inversely correlated to FPG (p = -0.25, p = 0.011), HbA1C (ρ = -0.29, p = 0.004), and GDS-H (ρ = -0.51, p < 0.001). However, no significant correlation was found with age, age at diagnosis of diabetes, duration of diabetes, BMI, systolic blood pressure (SBP), diastolic blood pressure (DBP), TC, TG, HDL-C, and LDL-C. The GDS-H score was found to be significantly positively correlated to FPG (p=0.25, p=0.012), HbA1C (ρ = 0.30, p = 0.002), and inversely correlated to HMSE score (p= -0.51, p < 0.001). However, no significant correlation was found with age, diagnosis, duration of diabetes, BMI, SBP, DBP, TC, TG, HDL, and LDL.

**Table 3:** Correlation analysis of various parameters with HMSE and GDS-H scores Statistically significant values are shown in bold. BMI: Body Mass Index, DBP: Diastolic Blood Pressure, FPG: Fasting Plasma glucose, GDS-H: Geriatric Depression Scale, HbA1C: Glycated Hemoglobin, HDL-C: High-Density Lipoprotein-Cholesterol, Hindi Mental State Examination: HMSE, LDL-C: Low-Density Lipoprotein-Cholesterol, SBP: Systolic Blood Pressure, TC: Total Cholesterol, TG: Triglyceride

Parameters	HMSE		GDS-H	
	Spearman's ρ	P-value	Spearman's ρ	P-value
Age	-0.19	0.065	0.04	0.675
Age at diagnosis of Diabetes	-0.07	0.474	-0.14	0.154
Duration of diabetes	-0.03	0.790	0.18	0.080
BMI	0.05	0.600	0.11	0.284
SBP	0.05	0.634	-0.01	0.930
DBP	0.08	0.425	-0.01	0.914
FPG	-0.25	0.011	0.25	0.012
HbA1C	-0.29	0.004	0.30	0.002
TC	-0.05	0.590	0.12	0.242
TG	-0.09	0.393	0.16	0.117
HDL	0.01	0.943	-0.02	0.828
LDL	-0.08	0.447	0.09	0.390
HMSE	--	--	-0.51	<0.001
GDS	-0.51	<0.001	--	--

The univariate binary logistic regression analysis was applied to calculate the odds of various factors on impaired cognition and depression (Table 4). We dichotomized the HMSE and GDS-H for binary logistic regression analysis. The chances of impaired cognition were found to increase with age, with an odds ratio (OR) of 1.1 (95% CI: 1.02-1.19, p = 0.013). Additionally, individuals living in rural areas had a higher likelihood of cognitive impairment than those in urban areas, with an OR of 3.25 (95% CI: 1.35-7.81, p = 0.009). Unmarried and widowed individuals had a higher chance of developing depression compared to married individuals, with an OR (95% CI) of 7.15 (1.46-35.09) (p = 0.015). The literate individuals were at increased risk of developing depression as compared to illiterate individuals with an OR (95% CI) of 3.23 (1.1-9.48) (p = 0.033). Increased FPG raised the odds of depression, with an OR (95% CI) of 1.01 (1.00-1.01) (p = 0.041). Higher HbA1C levels increased the odds of depression, with an OR (95% CI) of 1.39 (1.11-1.75) (p = 0.005).

**Table 4:** Univariate binary logistic regression analysis for impaired cognition and depression with various parameters Statistically significant values are shown in bold. HMSE: normal (score ≥ 24) and cognitive impairment (score < 24); GDS-H: normal (score: 0-9) and depression (score: 10-30). BMI: Body Mass Index, DBP: Diastolic Blood Pressure, FPG: Fasting Plasma glucose, GDS-H: Geriatric Depression Scale, HbA1C: Glycated Hemoglobin, HDL-C: High-Density Lipoprotein-Cholesterol, Hindi Mental State Examination: HMSE, LDL-C: Low-Density Lipoprotein-Cholesterol, SBP: Systolic Blood Pressure, TC: Total Cholesterol, TG: Triglyceride

	Impaired Cognition		Depression	
Parameters	OR (95% CI)	P-value	OR (95% CI)	P-value
Age (years)	1.1 (1.02-1.19)	0.013	1.02 (0.95-1.1)	0.586
Gender (Female vs Male)	0.66 (0.28-1.56)	0.348	2.19 (0.96-5)	0.063
Age at Diagnosis (years)	1.04 (0.98-1.09)	0.173	0.97 (0.92-1.02)	0.223
Duration of DM (years)	1.02 (0.96-1.08)	0.544	1.06 (1-1.12)	0.054
Family history (Yes vs No)	0.57 (0.25-1.31)	0.185	1 (0.45-2.23)	0.993
Hypertension (Yes/No)	0.70 (0.30-1.63)	0.405	1.24 (0.54-2.87)	0.610
BMI (kg/m^2^)	1 (0.92-1.08)	0.981	1 (0.93-1.08)	0.977
SBP (mmHg)	0.99 (0.97-1.02)	0.632	1 (0.98-1.03)	0.950
DBP (mmHg)	0.99 (0.96-1.03)	0.661	1.01 (0.98-1.05)	0.508
FPG (mg/dl)	1.01 (1-1.01)	0.067	1.01 (1-1.01)	0.041
HbA1C (%)	1.23 (1-1.53)	0.053	1.39 (1.11-1.75)	0.005
TC (mg/dl)	1 (0.99-1.01)	0.843	1.01 (1-1.02)	0.101
TG (mg/dl)	1 (1-1.01)	0.484	1.01 (1-1.01)	0.087
HDL (mg/dl)	0.99 (0.96-1.03)	0.784	0.99 (0.96-1.03)	0.765
LDL (mg/dl)	1 (0.99-1.01)	0.690	1.01 (1-1.02)	0.159
Diet (Non-veg vs Veg)	0.8 (0.35-1.8)	0.586	0.95 (0.43-2.11)	0.909
Smoking (Yes vs No)	1.03 (0.45-2.39)	0.941	0.48 (0.21-1.12)	0.090
Drinking (Yes vs No)	0.72 (0.23-2.28)	0.581	0.38 (0.11-1.26)	0.113
Religion (Hindu vs Muslim)	0.78 (0.28-2.16)	0.636	1.07 (0.39-2.94)	0.897
Residence (Rural vs urban)	3.25 (1.35-7.81)	0.009	1.79 (0.76-4.18)	0.181
Employment (unemployed & retired vs employed)	0.66 (0.27-1.64)	0.375	1.16 (0.47-2.85)	0.741
Social Status				
Upper Middle II	Reference		Reference	
Lower Middle III	0.46 (0.16-1.28)	0.137	1.26 (0.46-3.42)	0.651
Upper Lower IV	0.5 (0.18-1.38)	0.180	0.85 (0.31-2.32)	0.749
Education (Illiterate vs literate)	2.5 (0.89-7.06)	0.084	3.23 (1.1-9.48)	0.033
Marital status (unmarried & widowed vs married)	2.21 (0.62-7.82)	0.220	7.15 (1.46-35.09)	0.015

The multivariate binary logistic regression analysis with the forward conditional method was applied to determine the independent predictors of impaired cognition and depression. Entry criteria for the multivariate binary logistic regression analysis included variables with p-value ≤ 0.10 in the univariate binary logistic regression analysis (Table 4), and the results are presented in Table 5. The independent predictors of impaired cognition were age with OR (95% CI) of 1.13 (1.04-1.23), HbA1C with OR (95% CI) of 1.28 (1.02-1.61), and residence with OR (95% CI) of 1.28 (1.02-1.61). The development of depression in T2DM was forecasted by HbA1C with OR (95% CI) of 1.45 (1.14-1.85) and marital status OR (95% CI) of 9.50 (1.84-49.04).

**Table 5:** Predictors for impaired cognition and depression using multivariate binary logistic regression analysis Statistically significant values are shown in bold. Logistic regression analysis with a forward conditional method was used with an entry criterion of p≤ 0.10 and a removal criterion of p > 0.10. Input variables for impaired cognition: Age, Residence, Education, FPG, HbA1C. Input variables for depression: Gender, Duration of DM, Smoking status, Marriage status, FPG, HbA1C, TG. HbA1C: Glycated Hemoglobin

Cognition		
Parameters	OR (95% CI)	P-value
Age (years)	1.13 (1.04-1.23)	0.005
Residence (rural vs urban)	4.17 (1.57-11.08)	0.004
HbA1C (%)	1.28 (1.02-1.61)	0.037
Depression		
HbA1C (%)	1.45 (1.14-1.85)	0.002
Marital status (unmarried & widowed vs married)	9.50 (1.84-49.04)	0.007

A ROC curve analysis was performed after a multivariate binary logistic regression analysis of those continuous variables that were independent predictors for impaired cognition and depression (Table 6). For impaired cognition, age and HbA1C had an area under the curve (AUC) of 0.67 (p=0.002) and 0.65 (p=0.012), respectively. The optimal cut-off value for age was > 63 years with a sensitivity of 89.19% and specificity of 43.55%, while for HbA1C, it was > 7.7% with a sensitivity of 64.86% and specificity of 64.52%. For depression, HbA1c had an optimal cutoff value of 6.9% with a sensitivity of 81.40% and specificity of 53.57%, and the AUC was 0.69 (p <0.001).

**Table 6:** Cut-off points and diagnostic utility of continuous independent predictors HbA1C: Glycated Hemoglobin; AUC: Area Under the Curve

Parameters	Optimal cut-off value	AUC (95% CI)	P-value	Sensitivity (95% CI)	Specificity (95% CI)
Impaired cognition					
Age (Years)	>63	0.67 (0.58-0.77)	0.002	89.19 (74.6-97.0)	43.55 (31.0-56.7)
HbA1C (%)	>7.7	0.65 (0,54-0.74)	0.012	64.86 (47.5-79.8)	64.52 (51.3 – 76.3)
Depression					
HbA1C (%)	>6.9	0.69 (0.59-0.78)	<0.001	81.40 (66.6-91.6)	53.57 (39.7-67.0)

## Discussion

Cognitive impairment and depression are one of the most important health issues in the geriatric population because of their long-term implications and their effect on quality of life and increased dependency on family [4]. In the present study, the demographic, clinical, biochemical, and psychosocial parameters, along with cognitive function and depression, were explored in elderly T2DM patients. The findings from this cross-sectional study provide the prevalence of impaired cognition and depression, as well as the factors affecting them. This study provides deep insight to identify the high-risk population. The mental health status in T2DM patients is multifactorial in nature and depends on several factors and their interaction. Analysis of this study reveals that psychological, social, and glycemic factors interact to impact cognitive decline and depressive symptoms.

The prevalence of impaired cognition in our study was 37.37%, whereas other Indian studies had reported it to range from 16.9% to 50.5% [14-18]. Forty-three point four-three percent (43.43%) of participants were found to have depression in the present study, which is slightly higher in comparison to other Indian studies. They reported in the range of 22.8% to 41% [19-22]. A study from the USA reported the prevalence in the range of 2.0% to 28.8% [23]. A systematic review and meta-analysis reported that the prevalence of depression in T2DM patients was 28% globally, 32% in Asia, 24% in Europe, 27% in Africa, and 29% in Australia [8]. This difference in prevalence was due to variations in the study population, study tool, and different cut-off scores to define cognitive impairment and depression.

In the present study, the female group had significantly higher median GDS-H scores than the male group. A higher proportion of the female group exhibited depression as compared to the male (55.3% vs. 36.1%), though this difference is non-significant. In a meta-analysis, it was reported that the prevalence of depression was higher in diabetic females as compared to diabetic males (28.2% vs 18.0%) [24]. Another meta-analysis by Khaledi et al. (2022) also revealed a higher prevalence of depression in females as compared to males with T2DM [8]. The gender disparities in the prevalence of depression are multifactorial, involving poor social support, biological factors, and psychological aspects [21]. In our study, the difference is not statistically significant; this may be due to the small sample size and less numbers of females in the study.

In our study, rural patients demonstrated a significantly higher prevalence of impaired cognition (56.3% vs. 28.4%) and lower mean HMSE scores than urban patients. A rural residence increased the odds of impaired cognition by odds of 3.25 as compared to an urban residence. Patients residing in rural areas had higher median GDS-H scores compared to urban residents. However, a higher proportion of rural patients exhibited depression compared to urban patients (53.1% vs 38.8%), though this difference is non-significant. In a North Indian study, the prevalence of cognitive impairment in community-dwelling older adults in rural populations was 24.9% [25]. A study of older adults with T2DM in rural China reported a cognitive impairment prevalence of 50.22% [26]. A study on the South Indian population by Anugraha et al. (2022) reported that the prevalence of depression in rural and urban residents was 6.1% and 16.7%, respectively. However, this difference was not statistically significant [21]. A study in the North Indian population revealed that the rural subjects had a higher prevalence of depression than urban subjects (57% vs 31%), though this difference was marginally significant (p = 0.049) [27]. Rural and urban disparities may be due to differences in healthcare accessibility, socioeconomic status, delayed diagnosis, culture, lifestyle, and education deficits [27].

In our study, illiterate individuals had significantly higher median GDS-H scores, and a significantly higher percentage of illiterate individuals exhibited depression compared to literate ones (66.7% vs. 38.3%). Illiterate individuals had a higher likelihood of depression than literate individuals, with an odd of 3.23. The National Mental Health Survey (NMHS) of India investigated the relationship between literacy and mental health and reported a higher prevalence of depressive disorders in illiterate populations [28]. The association of illiteracy with higher rates of depression in chronic diseases like T2DM is multifactorial. The factors are lower socioeconomic status, less access to healthcare-related information, and lack of knowledge about disease management.

In the present study, unmarried and widowed patients had a higher prevalence of depression compared to married patients (81.8% vs. 38.6%), with an odds ratio of 7.15, indicating that these individuals were 7 times more likely to develop depression. The Longitudinal Ageing Study in India (LASI-2017-18) studied the prevalence of depression in subjects aged 60 years and above and reported a higher prevalence of depression among those who were widowed and living alone [29]. Another study by Murugan et al. (2023) on diabetic subjects showed a higher prevalence of depression in unmarried individuals than in married ones (81% vs 54%) [30]. In chronic diseases like T2DM, marital status is a key determinant of mental health. Unmarried and widowed individuals are at higher risk of developing depression due to social isolation, loneliness, and lack of emotional support. On the other hand, marriage is protective against the development of depression by providing emotional support and companionship.

In our study, HMSE scores were negatively correlated while GDS-H scores positively correlated with HbA1C and FPG levels, indicating that poor glycaemic control is associated with cognitive decline and increased depression. Additionally, HMSE and GDS-H scores were significantly inversely correlated, indicating that higher levels of depression are associated with poorer cognitive function. HbA1C and FPG are markers of long-term and short-term glycemic control, respectively. High HbA1C and FPG are linked to microvascular complications, oxidative stress, and neuroinflammation, which lead to neurotoxicity, resulting in cognitive decline and increased susceptibility to depression [5].

In the present study, the predictors of impaired cognition were age, HbA1C level, and residence while the predictors of depression were HbA1C level and marital status. Poor glycemic control, as indicated by higher HbA1C levels, is a significant predictor of both cognitive impairment and depression. Effective diabetes management and glycemic control not only affect physical health but also affect mental well-being. T2DM patients aged > 63 years and those with HbA1C > 7.7 and rural residences should be considered at the highest risk for cognitive impairment and prioritized for immediate screening for cognitive function. Unmarried or widowed patients with HbA1C > 6.9% should be prioritized for the screening of depression. These results indicate that elderly patients with type 2 diabetes mellitus, particularly those in high-risk groups, should be routinely screened for cognitive impairment and depression. Additionally, there is a need for comprehensive diabetes management programs.

This study has a few limitations: it is a cross-sectional study, has a small sample size, does not include a control group of non-diabetic elderly individuals, and is conducted in a single tertiary care center. Despite the absence of a control group, our study highlights the prevalence of cognitive impairment and depression in elderly T2DM patients, aiding early detection and intervention. This study lacks data on medication use, physical activity, and social support, which could impact cognitive impairment and depression in elderly T2DM patients. Further, multicentric longitudinal and case-control population-based studies should be conducted with large sample sizes in different ethnic groups; this is needed to validate the result of our study.

## Conclusions

In the present study, we found a higher prevalence of cognitive impairment and depression in the elderly type 2 diabetes mellitus subjects, emphasizing the significant mental health burden in this population. Our study also identified several factors associated with impaired cognition and depression. The illiterate, unmarried, and widowed had a higher prevalence of depression, indicating the role of education and social support in mental health outcomes. Patients older than 63 years with poor glycemic control, rural residence, unmarried or widowed, and illiterate should be screened for mental health status on a priority basis. Additionally, illiteracy emerged as a critical determinant, suggesting that educational status may play a vital role in both cognitive resilience and mental well-being. These findings point to the urgent need for targeted screening and early interventions in high-risk subgroups, particularly in resource-limited rural settings. Given the multifactorial nature of the observed mental health burden, a holistic approach involving regular mental health screening, patient education, and improved social support mechanisms should be prioritized in routine clinical practice.
